# ‘What price do you put on your health?’: Medical cannabis, financial toxicity and patient perspectives on medication access in advanced cancer

**DOI:** 10.1111/hex.13642

**Published:** 2022-11-06

**Authors:** Rebecca E. Olson, Alexandra Smith, Phillip Good, Morgan Dudley, Taylan Gurgenci, Janet Hardy

**Affiliations:** ^1^ School of Social Science University of Queensland St Lucia Queensland Australia; ^2^ Mater Health Services, Mater Research Institute University of Queensland Brisbane Queensland Australia; ^3^ Palliative Care St Vincent's Private Hospital Brisbane Brisbane Queensland Australia; ^4^ School of Medicine University of Queensland Herston Queensland Australia

**Keywords:** financial toxicity, medicinal cannabis, qualitative research, sociology

## Abstract

**Introduction:**

Following 2016 legislation permitting limited access to cannabis for research and medicinal purposes, the number of randomized clinical trials (RCTs) investigating the effectiveness of medicinal cannabis (MC) on symptom burden relief in cancer contexts has increased in Australia. This study aimed to understand the perceptions, hopes and concerns of people with advanced cancer regarding the future availability and regulation of MC in Australia.

**Methods:**

This qualitative study draws on semistructured interviews conducted between February 2019 and October 2020 in Brisbane, Australia, as part of an MC RCT substudy. Interviews were undertaken on 48 patients with advanced cancer in palliative care eligible to participate in an MC trial (*n* = 26 participated in an RCT; *n* = 2 participated in a pilot study; *n* = 20 declined). Interviews included a discussion of patients' decision‐making regarding trial participation, concerns about MC and perceptions of future availability, including cost. Transcribed interviews were analysed inductively and abductively, informed by constructivist thematic analysis conventions.

**Results:**

Overall, participants supported making MC legally accessible as a prescription‐only medication. Fear of financial toxicity, however, compromised this pathway. Steep posttrial costs of accessing MC prompted several people to decline trial participation, and others to predict—if found effective—that many would either access MC through alternative pathways or reduce their prescribed dosage to enable affordable access.

**Conclusions:**

These findings suggest that—despite a relatively robust universal healthcare system—Australians are potentially vulnerable to and fearful of financial toxicity. Prevalent in the United States, financial toxicity occurs when disadvantaged cancer patients access necessary but expensive medications with lasting consequences: bankruptcy, ongoing anxiety and cancer worry. Interview transcripts indicate that financial fears—and the systems sustaining them—may pose a threat to RCT completion and to equitable access to legal MC. Such findings support calls for embedding qualitative substudies and community partnerships within RCTs, while also suggesting the importance of subsidisation to overcoming injustices.

**Patient or Public Contribution:**

A patient advisory committee informed RCT design. This qualitative substudy foregrounds patients' decision‐making, perceptions and experiences.

## 1 INTRODUCTION

Patients with advanced cancer face numerous symptom burdens: pain, fatigue, nausea and sleep disturbance.^1^ Following 2016 legislation permitting limited access to cannabis for research and medicinal purposes in Australia,^2^ interest in the potential benefits of medicinal cannabis (MC) as an intervention for relief from symptom burden associated with cancer and advanced cancer has increased substantially.^3^, ^4^, ^5^ Several clinical trials have subsequently been initiated.^6^, ^7^, ^8^, ^9^, ^10^, ^11^, ^12^, ^13^ Data from trials, especially randomized controlled trials, however, can be ‘difficult to transfer to real‐life experiences’.^14^ While randomized clinical trials (RCTs) examine the effectiveness of MC at controlling symptom burden based on an experimental design, little is known about the experiences and concerns of Australians with advanced cancer considering MC.

Understanding patients' concerns, particularly related to access and regulation, is complicated by the history of cannabis as a recreational drug, and the funding, healthcare and regulatory practices specific to each country. Recreational cannabis (RC)—involving smoking or ingesting the cannabis plant which contains over 500 compounds—has been a prohibited substance for most of the 20th century.^15^ Recently, cannabis has been progressively remedicalized as a viable treatment for a range of illnesses, conditions and symptoms,^16^ typically involving the specific chemical compounds cannabinol (CBD) and tetrahydrocannabinol (THC) in isolation or combination.^17^ This remedicalization has occurred on a global scale, with legitimate channels for accessing MC now established in North America, South America, Europe, Israel and Australia.^18^ How receptive countries have been to MC, however, varies. Jamaica's legal therapeutic cannabis market, for example, faces diplomatic and marketing challenges, with constraints imposed by agreements with the United Nations and the United States and purchasers conflating RC and MC.^19^ Stigma has also been found to be a barrier to accessing MC in the United States,^20^ and a contributor to perceptions and experiences of MC use in Thailand^21^, ^22^, ^23^ and Canada,^24^ but does not feature as such in the limited Australian‐focussed scholarship.^5^, ^25^

In Australia, MC users face two tiers of regulation. Cannabis policy is split between federal and state jurisdictions, with federal policy progressing slowly, and states devising their own approaches.^26^ Despite 2016 legislation changes allowing limited access to MC via prescription from strictly regulated healthcare specialists, survey research suggests Australians still access cannabis through illicit channels and hold concerns about financial and administrative barriers to accessing MC within existing regulatory frameworks.^27^ This research suggests that 62.6% of Australians assessed MC as prohibitively expensive, and 87.3% found the existing regulatory framework difficult to negotiate.^27^ The number of Australians accessing MC has progressively increased, with a total of 159,665 approvals issued by 31 August 2021, 82.4% of which were issued after January 2020.^28^

Concerns about access must be contextualized with reference to Australia's healthcare system: a mixed public–private system underpinned by Australia's universal coverage known as Medicare.^29^ Under Medicare, costs associated with hospital‐based and some community‐based care (e.g., bulk billing General Practices) are funded through taxation (e.g., Medicare Levy; Medical Levy Surcharge).^30^ Australians are, however, incentivized through tax deductions to supplement with private health insurance,^31^ and approximately 46% do so, allowing them access to private hospitals and ‘extras’ coverage, including dental, optical, allied health and other services.^29^, ^32^ Within this system, Australians access prescription medication at a reduced cost as most are included on the government's Pharmaceutical Benefits Scheme (PBS). For patients, this scheme dramatically reduces the price of pharmaceuticals filled through a pharmacist, requiring only modest out‐of‐pocket co‐payments. Such co‐payments are capped at $42.50 AUD for each PBS medicine dispensed and $6.80 AUD for those with concession cards (e.g., pensioners, students), and cumulatively at $1542.10 AUD or $326.80 AUD annually.^33^ In this same environment of government‐subsidised medicines, however, authorized MC products cost consumers between $350 and $600 per 100 ml (oil) and around $200 for a 15 ml spray (figures accurate as at August 2022).

### 1.1 Objectives and theoretical framework

Survey research suggests Australians are concerned about MC's financial and administrative burden,^27^ but little to no in‐depth research has been conducted with Australians with advanced cancer. This is a unique population with incurable, but often long‐term disease, who are underrepresented in clinical trials and research more generally—and especially so those with poor performance status and/or high symptom burden.^34^, ^35^, ^36^, ^37^ This study aims to understand the perceptions, hopes and concerns of people with advanced cancer regarding the future availability and regulation of MC in Australia.

Supporting this objective, we draw on a concept of growing interest in cancer care—financial toxicity—extended by a social constructionist understanding of medication use as situated and agentic. Financial burden has traditionally been understood in terms of the direct financial costs associated with treatment, such as out‐of‐pocket expenses remaining after government subsidy for certain medications, or the costs associated with attending multiple clinicians across several specialist clinics.^38^ Financial toxicity represents growing recognition of the need to broaden conceptualizations of financial burden to account for indirect costs such as the associated emotional burden and the coping strategies patients employ.^38^, ^39^

Financial toxicity occurs when cancer patients—especially those with early and more severe disease—pay out‐of‐pocket costs (including travel and accommodation) to access necessary but expensive interventions, often while experiencing income loss due to reduced hours or early retirement, with lasting consequences to their finances and mental health, including debt, bankruptcy, emotional well‐being (distress, anxiety and worry about a recurrence), quality of life and survival.^39^, ^40^, ^41^, ^42^ Unsurprisingly, financial toxicity is more common in countries where healthcare is predominantly privately funded; 53.7% of cancer patients surveyed in the United States reported experiencing financial toxicity.^39^ It is less prevalent in Australia, with research suggesting its commonality to be near 7% for Australians 12 months postdiagnosis with colorectal cancer, compared to 39% for patients with colorectal cancer in Ireland,^40^ and 20% for Australian men with prostate cancer.^43^

Although financial toxicity represents a broader conceptualization of financial burden, with terms like ‘cost‐related nonadherence’ used to describe strategies of coping with financial toxicity,^39^ the concept can be critiqued as furthering a clinician‐centred understanding of financial burden. Thus, we expand our conceptual framework, drawing on Conrad's^44^ classic medical sociology concept of ‘medication practice’, helping us to shift our focus towards a patient‐centred understanding of MC's financial and regulatory availability for Australians with advanced cancer. Medication practice can be defined as, ‘how people manage their medications, focusing on the meaning and use of medications’ and viewing ‘patients as active agents rather than passive recipients of doctors' orders’.^44^ Taking such an approach allowed us to prioritize a patient‐centred examination of concerns and hopes regarding MC's future availability, to inform justice‐oriented^45^ RCT study design and policy.

## 2 METHODS

### 2.1 Study design and recruitment

This qualitative substudy examined the perceptions of people with advanced cancer eligible to participate in an MC trial.^25^ Semistructured interviews were arranged with recognition of the time and communication needs of people with advanced cancer, taking a pace set by the interviewee to accommodate for any fatigue. Compared to surveys, interviews allowed for the collection of richer, inductive findings into subjective experiences and concerns about MC's future availability.^46^ An experienced qualitative researcher oversaw data collection, with interviews facilitated in Brisbane, Australia, between February 2019 and October 2020. The substudy was approved by Human Research Ethics Committees at two hospitals: the Mater Hospital (HREC/17/MHS/97) and St Vincent's Hospital (HREC 17/27).

To participate in interviews, participants had to be eligible to consent to one of three MC trials conducted by the research team; the protocols for the two RCTs and results for the pilot study have been published.^6^, ^7^, ^8^ Relevant eligibility criteria for these MC trials included the following: (a) having an advanced (incurable) histology‐proven cancer diagnosis as defined by its anatomical components as locally advanced or metastatic; (b) receiving palliative care at the treating hospital; (c) experiencing symptom burden; (d) being aged 25 or older.^6^, ^7^, ^8^ MC was sourced through a registered MC manufacturer and made available to those participating in an MC trial through a hospital pharmacy, dispensed as an oil.^6^, ^7^, ^8^ Recruitment for interviewees, led by the clinical trials coordinator, co‐occurred with RCT recruitment. Purposive sampling enabled balanced representation across the two interviewee groups—those who declined and those who consented to MC trial participation—and in terms of gender and age (see Table 1).

**Table 1:** Demographic characteristics of interview participants

Characteristic	Interview participants		
	Trial participant (*n* = 28)	Declined trial participation (*n* = 20)	Total (*n* = 48)
Gender, *n* (%)			
Male	12 (25)	11 (22.91)	23 (47.91)
Female	16 (33.33)	9 (18.75)	25 (52.08)
Age in years, *n* (%)			
≤49	3 (6.25)		3 (6.25)
50–69	15 (31.25)	9 (20.8)	24 (50)
70–89	10 (20.83)	11 (22.91)	21 (43.75)
Marital status, *n* (%)			
Married/civil partnership	23 (47.91)	11 (22.91)	34 (70.83)
Divorced/separated/widowed	5 (10.42)	8 (16.66)	13 (27.08)
Single		1 (2.08)	1 (2.08)
Ethnicity, *n* (%)			
Anglo‐Saxon/English	17 (35.41)	18 (37.5)	35 (72.92)
Australian	3 (6.25)		3 (6.25)
Pacific Islander	3 (6.25)		3 (6.35)
Australasian	1 (2.08)		1 (2.08)
Scottish	1 (2.08)		1 (2.08)
Undisclosed	3 (6.25)	2 (4.16)	5 (10.41)
Education level, *n* (%)			
Did not complete high school	3 (6.25)		3 (6.25)
High school	24 (50)	16 (33.33)	40 (83.33)
Bachelor's degree		1 (2.08)	1 (2.08)
Unknown	1 (2.08)	3 (6.25)	4 (8.33)
Primary cancer diagnosis, *n* (%)			
Breast	6 (12.5)	6 (12.5)	12 (25)
Prostate	3 (6.25)	7 (14.58)	10 (20.83)
Lung	4 (8.33)	3 (6.25)	7 (14.58)
Ovarian	3 (6.25)		3 (6.25)
Endometrial	3 (6.25)		3 (6.25)
Urothelial		2 (4.16)	2 (4.16)
Pancreatic	2 (4.16)		2 (4.16)
Colorectal/rectal	2 (4.16)		2 (4.16)
Bladder	1 (2.08)		1 (2.08)
Bile duct	1 (2.08)		1 (2.08)
Gastrooesophageal	1 (2.08)		1 (2.08)
Glioma	1 (2.08)		1 (2.08)
Kidney		1 (2.08)	1 (2.08)
Mesothelioma	1 (2.08)		1 (2.08)
Unknown primary		1 (2.08)	1 (2.08)

### 2.2 Data collection

Interviews lasted between 20 and 60 min, facilitated by one of two experienced interviewees with backgrounds in sociology and social work. Most interviews (*n* = 42) were face‐to‐face, held within a hospital consultation room in a quiet area of the hospital. Following public health measures related to COVID‐19, interviewees were given the option—in accordance with an approved ethics amendment—to participate via telephone. Six interviews were subsequently conducted via telephone. Using an interview guide, facilitators prompted participants to reflect on their perspectives on MC and research, their main reasons for participating or not participating in a trial, their perceptions on current and changing MC laws and their opinions on future access. Following an iterative approach to data collection, data generated in earlier interviews refined the focus of the interviews^47^; themes identified in initial analysis informed revisions to the semistructured interview guide. For example, in considering transcripts from initial interviews, several participants described financial barriers, prompting us to add a question about financial concerns to the interview guide. All interviews were audio‐recorded, with each interviewee being assigned a numerical pseudonym following verbatim transcription.

### 2.3 Data analysis

Data analysis was guided by constructivist approaches to thematic analysis.^48^, ^49^, ^50^ Grounded theory informed elements of the study design, such as taking an iterative approach to data collection and analysis.^47^ However, in line with the epistemological positioning and appreciation of knowledge as co‐constructed^51^ that underpins constructivist thematic analysis, the research team's reflexive and theory‐informed positioning was foregrounded (rather than bracketed) to prioritize inductive and abductive analysis.

Transcripts were analysed thematically and by case, using NVivo 12 qualitative analysis software to support data management, and to create and organize codes based on the research aims, interview questions and evolving findings. Open coding was undertaken to identify new themes and facilitate comparison. Regular meetings across the team of qualitative researchers and RCT study leads in the early stages of data collection, supported our discussion of findings from selected transcripts. Such meetings prompted us to attend to our differing positions in engaging with transcripts and foreground multiplicity in our theory‐informed interpretations, as we come from disciplinary backgrounds in sociology, medicine, social science, social work and anthropology. Following the discussion in our meeting on findings related to cost, themes were mapped against scholarship on financial toxicity,^39^ and extended by Conrad's^44^ concept of medication practice. Informed by these discussions, two members of the research team progressed data analysis and code development.

## 3 RESULTS

A total of 48 people with advanced cancer were recruited: 26 who agreed to participate in an RCT, 2 who agreed to participate in a pilot study and 20 who declined RCT or pilot study participation (see Table 1). Participants were relatively evenly divided in terms of gender (52% female) and age (50% aged 50–69).

**Figure 1:**
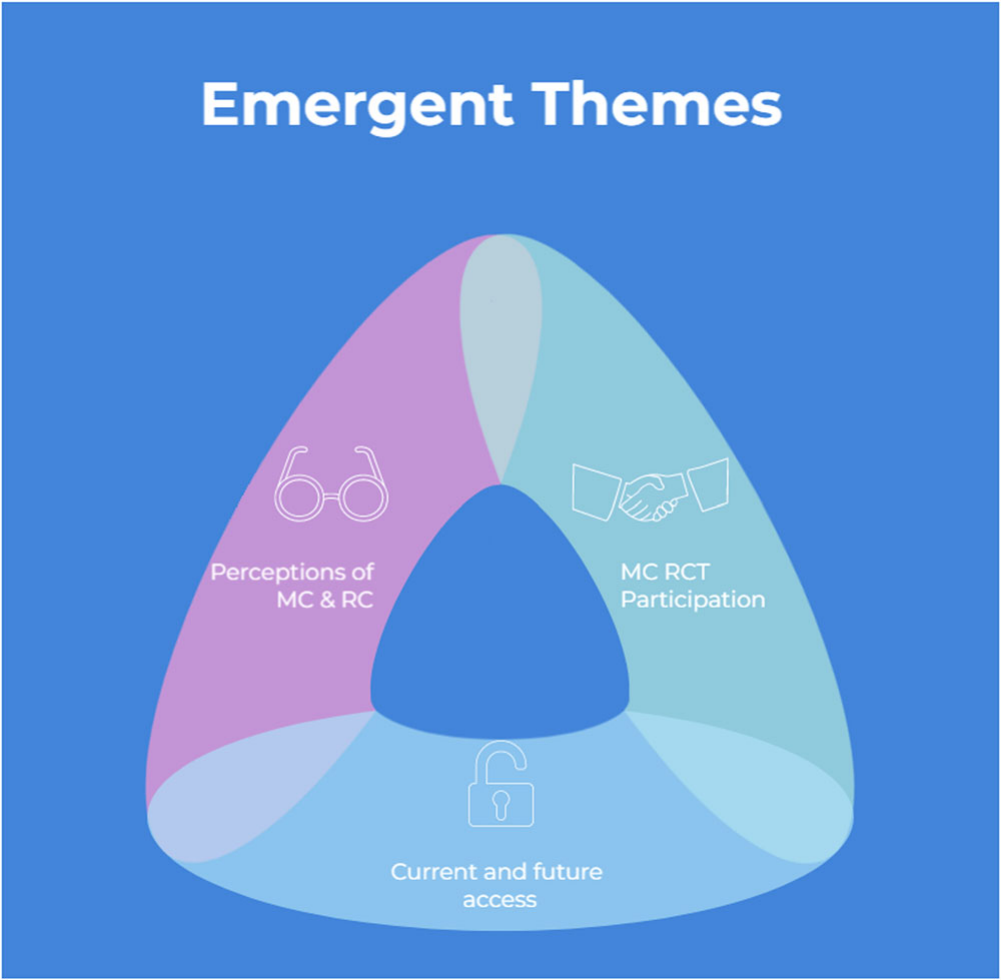
Summary of emergent themes

Several themes were produced through our analysis(see Figure 1). This section first presents themes on patients' perceptions of how MC should be made accessible in the future, with most supportive of restricting access to a prescription‐only medication, dispensed by a pharmacist. We then provide patients' perceptions on current barriers—financial and administrative—to this accessibility, with many expressing concern about the high cost of accessing MC and predicting access outside of pharmacies to manage these costs.

Themes are supported by data displays from patient interviewees, including reference to their participant number (e.g., ‘P5’ for participant 5), gender (e.g., ‘F’ for female), age (e.g., 70s) and trial participation status (e.g., ‘declined’ indicates they did not participate in an MC trial; ‘Pilot‐CBD’ indicates participation on the MC Pilot study^7^ receiving the CBD intervention). Information is provided as to which RCT arms participants were assigned (e.g., MC1‐CBD refers to the intervention arm of the MC1 study^6^; MC1‐Placebo refers to the control group). It is also noted when this information is not yet available (e.g., MC2‐Blinded refers to participation on the MC2 study,^8^ where information on control and intervention group assignment remains blinded). Where participants described sourcing cannabis for medicinal purposes outside of the trial, this is indicated (e.g., Non‐trial MC).

### 3.1 Future access: Who and how

Interviewees were supportive of making MC accessible, to ‘people who need it’ (P26, F, 50s, MC2‐Blinded) and who were ‘going to be responsible’ in using it (P38, F, 70s, Non‐trial MC). One interviewee went so far as to suggest a screening process:

[J]ust as long as … people that actually need it are screened properly and have all the documentation necessary to actually go on it. Because we see too many people who don't really need it, access it and then they screw it up for the rest of us. (P25, F, 30s, MC2‐Blinded)

Although a minority said ‘it should be open to everyone and anyone’ (P14, F, 40s, MC1‐CBD), other participants specified that it should be available to populations where other interventions have been shown to be less effective, such as chronic pain and cancer care (P21, F, 30s, MC1‐CBD), terminal illness (P18, M, 60s, MC1‐CBD) and conditions such as Parkinson's disease:

[It should be made available for] I suppose mainly—there are a lot of cancer sufferers. For anyone really that's suffering, with people that [have] Motor Neurone Disease, if it helps them. People that have multiple sclerosis, if it helps them. Parkinson's, all of these really challenging diseases. If it helps those people, then I believe that they should be able to have access to it. (P23, F, 70s, MC1‐Placebo)

In discussing how MC should be made available, one interviewee saw too much regulation as problematic: ‘I don't think it should be controlled by state or federal government. I think it's something that they don't need to be involved in’ (P18, M, 60s, MC1‐CBD). Others (P26, F, 50s, MC2‐Blinded; P29, M, 60s, Non‐trial MC) suggested RC should be decriminalized and MC made available at pharmacies.

[I think it should be available] freely, over the counter. Yeah … I think it should be decriminalised. There shouldn't be criminal convictions and all the rest because you've got a bit of pot in your pocket. (P29, M, 60s, Non‐trial MC)

Most, however, responded by referencing existing mechanisms for regulating, supplying and dispensing medicines in the Australian context. Participants concluded that availability should be ‘controlled’ (P14, F, 40s, MC1‐CBD; P37, M, 66, declined), to prevent MC from being ‘abused’ (P33, F, 70s, declined) and out of a concern for safety (P13, F, 70s, MC1‐Placebo).

If I can't get it on a prescription I wouldn't be having it, no way, no. So, it's got to be something that you've got to get…. through chemists, through your doctor and you must have to have a script for it at all times. (P15, M, 70s, MC1‐Placebo)

Accordingly, several participants proposed limiting access to those with a prescription made by a specialist (P13, F, 75, MC1‐Placebo) or General Practitioner (P12, M, 60s, MC1‐Placebo; P22, M, 70s, MC1‐Placebo; P33, F, 70s, declined; P18, M, 60s, MC1‐CBD).

Just from your doctor and the same as any other medicine is dispensed … A prescription, yes. I don't think you could just walk in and buy it. It would still have to be controlled. (P37, M, 60s, declined)

Even those participants with a history of sourcing MC through alternative pathways, and who decried the challenges of accessing MC for others, talked about controlling the supply of MC to mitigate perceived abuse:

You've got to put some controls on it otherwise you'll get abuse…. if you just say, ‘oh you're feeling depressed and so you can get an [unclear]’, well 98 per cent of Australia would be depressed in the morning … it would need to be controlled, there's no doubt about it … possibly the same way as you have the prescription medication. (P30, M, 60s, Non‐trial MC)

Overall, most participants saw benefits to limiting access to MC to individuals with a diagnosed condition and a medical prescription, ordered by a physician and dispensed by a pharmacist. Financial and administrative challenges, however, were said to pose barriers to achieving the perceived safety and hoped‐for equity of regulated access to MC.

### 3.2 Barriers to access: Cost

In discussing future access to MC or in response to questions specifically about any financial concerns, 29 interviewees (17/28 on an MC RCT; 12/20 who declined RCT participation) described financial concerns, especially pensioners. Interviewee P13 (F, 70s, MC1‐Placebo), for instance, said ‘$400 is a lot for a little bottle’, and Interviewee P39 (M, 70s, declined) described MC as ‘cost prohibitive for a pensioner’. Interviewees described their worries regarding the projected cost of accessing MC outside of RCTs, and the ways in which government regulatory control might overcome these barriers.

Five interviewees saw the posttrial cost of accessing MC as prohibitive to RCT participation, among other barriers,^25^ directly citing cost as an impetus behind their decision to not participate in an MC RCT. Interviewee P31, for instance, said ‘No, the reason I didn't take part in the trial was the fact that [sighs]—in the end I'm not going to benefit from it because it's going to be too expensive’ (F, 60s, declined). Interviewee P45 (F, 60s, declined), similarly declined to participate in the RCT, saying, ‘I know the trial was free, but the cost, yeah, afterwards. So, I thought, ooh and being on a pension, it would take quite a bit of that’. Interviewee P37 also cited cost in their decision to decline participation: ‘I was told, when I first started talking about the trial, that if I felt there was benefit, that I could actually stay on medicinal cannabis. However, the costs were very high’ (M, 60s, declined).

Raising questions of justice around equity of access, two participants asked about the merit of conducting the RCTs if the intervention was not going to be accessible.

I was told that … it was going to be three or four hundred [dollars] a [pop] to get—have it. Just—that's out of the reach for a lot of people. Why are they even doing it if we're not even going to be able to afford to have it? It just seems crazy. (P31, F, 60s, declined)

[W]hat are we trying to do here? All this study and all this research and trials and everything and people are taking the right drug, but then can't afford to take it. (P14, F, 40s, MC1‐CBD)

These interviewees positioned lack of affordable access as a central drawback to the trial: ‘the main danger is it would become too expensive for the average person who really needs it’ (P38, F, 70s, declined).

Others posed the cost as a concern, but not a deterrent to trial participation. One interviewee, a retired General Practitioner, stated explicitly that cost was not a constraint. ‘We are quite comfortable financially…. My wife would … spend whatever it takes, I know that, without the slightest hesitation, to improve the quality of my life’ (P44, M, 80s, declined). Rather than cost, symptom‐related fatigue posed a barrier to his participation. For others, cost was concerning: ‘Once this [trial participation has ended] I don't really know whether I would go further because I've been told that it's very expensive’ (P15, M, 70s, MC1‐Placebo). The disparity in access prompted Interviewee P8 to lament this financial injustice:

[I]t should be affordable for everybody. There's no reason why it shouldn't be whether you're rich or poor or whatever … that's where I think the gap is. People when they've got all the money can get it and people that don't are ones that don't even know where to start with it basically. (P8, F, 50s, Pilot‐CBD)

Fears of financial difficulty prompted many to deliberate on the cost of the intervention, their need for the intervention, the challenges of financing their posttrial prescription and the consequences of prioritizing MC over other needs. Interviewee P23, for example, positioned health and cost as competing priorities:

I do have concerns. We are only pensioners. But what price do you put on your health? If I feel that it has been beneficial to me and I'm feeling better on it, then to me, that's priceless. (P23, F, 70s, MC1‐Placebo)

Another positioned MC and its cost as like an illicit ‘drug habit’, jokingly illustrating the impact of the pharmaceutical on their budget:

After this trial it's going to cost me a fortune and none of it's subsidised, is it? … Apparently, it's around $400 a bottle of what I've got. The little tiny ones. I'd be using that quite a bit so that's a bit scary. Yeah, that's a pretty heavy drug habit [laughs]. (P24, M, 50s, MC1‐CBD)

Upon deliberation, these patients concluded that their health should be the priority. Interviewee P18 was less certain, wary of the risk that MC posed to their budget.

The costs and affordability, yeah … it's one of those things that you've just got to deal with that if—there's ways of finding money to get help, to get funding and that type of thing. Sometimes it's easy, sometimes it's not. Yeah, you've got to consider cost. You have to. (P18, M, 60s, MC1‐CBD)

Such deliberations assumed that MC was beneficial. However, not all participants were sure of MC's efficacy. For these interviewees, cost concerns were compounded with worries that they were wasting money—‘just throw[ing] it down the drain’ (P9, M, 70s, MC1‐Placebo)—on a medication unproven to address their symptoms:

Oh, it sounds expensive to me at $100 a week on something that may or may not work. I guess $100 a week wouldn't be a major worry financially, it just seems like a waste for something that I feel doesn't suit my circumstances. (P39, M, 70s, declined)

The quotes presented above relay interviewees' shock and fear—using words like ‘ludicrous’ (P14, F, 40s, MC1‐CBD) ‘crazy’ (P31, F, 60s, declined) and ‘scary’ (P24, M, 50s, MC1‐CBD)—related to the cost of accessing MC posttrial. Positioning varied, with some having the means to pay, others turning down participation in a trial due to the posttrial cost, and others still—many pensioners—uncertain, caught between prioritizing the cost of attending to their symptom burden through (not yet proven) MC prescriptions and affording other living costs. Without government regulation and subsidy to reign in the cost, interviewees predicted that patients would reduce their intake or access MC via alternative pathways.

### 3.3 Overcoming financial and administrative barriers: Stretching and shifting

To manage the high cost of accessing MC posttrial, and reap the expected benefits, several interviewees described stretching and shifting: making a vial last longer by taking less or changing to alternative pathways. Interviewee P45, for example, described a patient who found MC effective in controlling symptoms, but reduced their MC doses because of financial constraints:

I knew of a friend who did actually buy the medicinal marijuana … because she had really, really bad back pain. It did help her, but it was very expensive. So, she went down to only having half the dose to make it stretch a bit more, but she found it worked amazingly. (P45, F, 60s, declined)

Others considered shifting, accessing MC through other (in some cases, illegal) means, such as via the internet (P42, F, 60s, declined) or black market (P26, F, 50s, MC2‐Blinded; P32, M, 60s, declined). Interviewee P11 described this in economic terms:

I'd [access MC] through the legal channels, but if I was sitting here and I didn't have—and the legal channels were $1000 a day, and the illegal channels were $10 a day, and I didn't have the money, then I'd obviously go that way. Do you know what I mean? It all depends on the person's capacity to pay. (P11, M, 60s, MC1‐Placebo)

Interviewee P10 emphasized both the financial and administrative ‘rigmarole’ as deterrents to accessing MC legally:

Because it's too expensive if they buy it. Because they have to go through too much of a rigmarole to get registered to take it. Then they have to pay exorbitant amounts and then they have to renew that every six months. So, it's a lot for people…. I think I probably would know more people who would access it the other way. (P10, F, 60s, MC1‐CBD)

Interviewee P40 similarly implied that the current financial and administrative ‘speed bumps’ associated with legally accessing MC were likely to push many towards unregulated avenues:

Well, I don't think you can ever agree with the black market or even going, buying it online, I don't agree with any of that. But having said that the only way that's ever going to be stopped is for our system to make it available, a lot easier, without putting all the speed bumps in the road. (P40, M, 80s, declined)

Although this interviewee—like many others—didn't ‘agree with’ shifting to alternative MC access pathways, others showed less reservation. In such interviews, a common thread of pragmatism, and a related lack of stigma, was evident in discussions of accessing MC outside regulated pathways. Interviewee P13, for example, discussed her current experience in obtaining MC where her main concern was about trust in the quality and safety of the MC accessed via an alternative pathway:

I was looking where I could get the oil and I put it on social internet and someone came forward and said yes, I've been on it …. I can get the oil for you…. The thing that stopped me was the price … I didn't get it from there, but I … rang around all the naturopath people to see if they had it or knew where I could get it. This lady came forward so I just got the cannabis stuff, the marijuana, and she made the oil for me. I trusted her because it helped her … I wouldn't buy it from anybody that I didn't know…. the lady that made it up for me she was okay too. But it's dicey if you just go straight there and don't make inquiries or anything. (P13, F, 70s, MC1‐Placebo)

Even interviewee P14, a police officer, viewed accessing MC via alternative pathways pragmatically:

If you can go to [town] and get it, I don't know, $50 or whatever, I don't know, but compared to $200 for every three days. Who would begrudge anyone to do that? As a police officer, yes, I would still charge them, but I can tell you the courts will get sick of it, because that's what we'll be putting in the paperwork. (P14, F, 40s, MC1‐CBD)

Despite interviewees' overwhelming support for restricting MC access to a pharmacy prescription, financial and administrative barriers posed a threat to this pathway's viability. Interviewees predicted that—without regulation and subsidisation—patients would likely stretch, altering prescribed dosages to improve affordability, or shift, unashamedly sourcing MC via alternate pathways.

### 3.4 Overcoming financial barriers: Government regulation

To facilitate smoother access—financially and administratively—interviewees, especially those on a pension or reduced income, overwhelmingly suggested government regulation through the PBS.

Because I'm on a pension and I live from week to week, so I can't afford to pay much…. if it gets to be on the PBS … then I really can afford it. (P3, F, 60s, Pilot‐CBD)

It should go on the PBS. It'd make it so much easier for so many people…. We're pensioners. We'll find the money, but it's going to be a bit hard. (P13, F, 70s, MC1‐Placebo)

Some suggested government regulation through the PBS ‘so that more and more people can get the benefits of it’ (P22, M, 70s, MC1‐Placebo): as a matter of equity and justice for patients in need of medication.

Through the PBS. Yeah. Definitely. They do so much for everybody else. Why not for these people that really need it? (P19, F, 50s, MC1‐Placebo)

So, the costs for the people who are falling within the categories for its use who want to use it and find benefit from them, it should be made financially within their reach under a PBS‐type subsidised scheme and it shouldn't be to their detriment if it's providing them health and pain relief and assistance in coping with their medical health or mental health. (P34, M, 50s, declined)

Others positioned PBS regulation as symbolic of MC's efficacy, if proven, just like any other evidence‐based pharmaceutical intervention.

If it's got proven benefits, it should be on the PBS. (P39, M, 70s, declined)

[It should be made available through the PBS … if you've got something that it can help with, like Endone or something like that—that helps—well then you should be able to access that the same sort of way. (P31, F, 60s, declined)

To improve affordability, equity of access for patients and equity in MC's treatment as a pharmaceutical intervention—interviewees supported making MC a prescription‐only medication subsided by Australia's PBS.

## 4 DISCUSSION

This qualitative study aimed to understand the perceptions, hopes and concerns of people with advanced cancer regarding the future availability and regulation of MC in Australia. Overall findings suggest that patients are supportive of making MC legally accessible as a prescription‐only medication. Fear of the financial risks, however, compromised this pathway. The administrative ‘speed bumps’ and steep posttrial cost of accessing MC prompted several people to decline trial participation, and others to predict—if found effective—that many would either reduce their prescribed dosage to enable affordable legal access, or access MC through alternative pathways. Below, we discuss this contribution, theorizing the financial risks of accessing MC posttrial as financial toxicity, and explicating the threat it poses to equitable access to legal MC and RCT participation. We then consider the implications of this finding for policy and RCT design, suggesting subsidisation and qualitative substudies as ways of foregrounding and overcoming possible injustices.

Interviewees were overwhelmingly supportive of making MC legally accessible as a prescription‐only medication. As 28 of our interviewees were individuals with advanced cancer consenting to participate in trials—a hypermedicalized context involving a high degree of medical control, including, in these trials, restricting eligibility to those with no cannabis in their system—this sample may seem to be providing a relatively skewed perspective. However, other Australian research examining broader public perceptions of MC use suggests that these patients/participants are not outliers—acceptability of MC is high amongst the general population^27^, ^52^ and a majority of general practitioners are also supportive or neutral on MC use.^53^ Furthermore, it is important to note that patients were screened for RCT eligibility after consenting to participate. Thus, some interviewees who consented to participate in an MC RCT and interview, may have been found to be ineligible later because of having cannabis in their system. Despite support for it, MC was also perceived as a current or potential source of financial toxicity by patients with advanced cancer interviewed for this study—amongst those who consented MC RCT participation and those declined—with five participants directly citing cost in their decision to not participate in an MC RCT. Said another way, MC was perceived by many as a necessary or potentially necessary intervention, with associated costs that could prompt financial strain.^39^ Many reflected on the posttrial cost of accessing MC, using emotional and disparaging language to express their fear and concern. This finding supports research from the United Kingdom^54^ and Canada^55^ showing significant financial barriers to accessibility for patients, despite MC being available in these countries within regulatory frameworks. However, it may be surprising in the Australian context, given that financial toxicity is less prevalent in this country,^40^ especially compared to countries with limited public healthcare systems, such as the United States.^39^ This finding may also be surprising considering financial toxicity is often associated with an early‐stage diagnosis.^40^ Nonetheless, financial toxicity was a concern for interviewees with advanced cancer in this study. Many interviewees were facing chronic symptom burden and were pensioners, with few classified as high socioeconomic status: all factors which have been found to be significant predictors of a financial burden and financial toxicity.^38^, ^56^

In responding to their financial toxicity concerns, interviewees described several mitigation strategies: stretching, shifting and declining. Some participants predicted ‘stretching’ their supply to better weather MC's posttrial financial imposition, taking less than the recommended dose to reduce their weekly MC expenditure. This is a well‐known strategy for coping with financial toxicity, referred to as ‘cost‐related medical nonadherence’^39^ or ‘cost‐related medication underuse’^57^ within medical scholarship and, less pejoratively, active or agentic ‘medication practices’ within sociological scholarship.^44^

Shifting—to alternative markets—was another financial toxicity coping strategy, but less acknowledged within cancer scholarship and potentially unique to MC. Despite overwhelming support for restricting access to a pharmacy prescription, interviewees described unreservedly sourcing MC via less than legal pathways. Blurring or hybridizing RC and MC, some predicted or actively engaged in abandoning concerns related to safety and control, and sourcing uncompounded cannabis online or via a trusted supplier for a fraction of the cost and without the administrative burden. This finding suggests that pragmatism in the Australian context may override the stigma related to accessing MC found in research from the United States.^20^ It also supports research by Mahamad and Hammond^55^ pointing to the continued existence, and indeed flourishing, of ‘black market’ sources of medicinally used cannabis in environments of legalized, regulated MC. Within the context of financial toxicity,^39^ this study draws attention to the ‘coping’ practice of sourcing medication illegally to treat their conditions—a practice suggested to be widespread but below the ‘public gaze’.^15^ Sociologically, this practice is referred to as engaging in ‘covert’ or ‘subaltern’ therapeutics: using interventions deemed outside of medicine, resistant to biomedicine (such as ‘folk medicine’), or, in the case of marijuana, criminalized.^15^ For policy, this finding raises important questions about state processes with poor streamlining, potentially posing a threat to MC schemes,^19^ and certainly motivating potential MC users' consideration of less legal competitors.

In addition to stretching and shifting, declining was a further strategy for mitigating MC's perceived financial toxicity. Five interviewees declined to participate in an MC trial citing cost as a reason. Despite MC being available at no cost to trial participants, the high posttrial cost prompted these interviewees to circumnavigate financial concerns by avoiding MC altogether.^25^ This barrier to MC RCT participation raises important concerns about equitable access to tested interventions, and the potential impact of these concerns on patient decision‐making regarding trial participation. In her research on disparities in RCT participation, Fisher^58^ shows marginalized men overrepresented in early‐stage pharmaceutical testing, but underrepresented as intervention users. Our study suggests economically disadvantaged participants may be deterred from participating. Such inequities could undermine RCT completion, as well as impact fair and equitable access to tested interventions following trial completion. While recruitment was not an issue for the MC trials supported by this qualitative substudy, it is a common problem. An estimated 50% of RCTs fail to recruit to their targets,^59^ a problem amplified within palliative care contexts, where sample attrition is a regular and expected occurrence.^60^

Qualitative substudies—as illustrated through this study—and community partnerships can foreground inequities that threaten to undermine RCT recruitment. Fortuna et al.^45^ suggest countering the reductionism that underpins the scientific method—epitomized by RCTs—with humanistic approaches—such as qualitative and participatory methods—that ‘prioritize[] the human experience and promote[] the inclusion of disadvantaged populations as partners in research’. As evidenced in this study, ‘methodological pluralism’^45^—through a qualitative substudy—can allowed researchers to identify differences in power and resources that could undermine clinical research and clinical outcomes.

While small revisions to study designs and research practice can go some way towards attending to inequities, broader change is also needed. Findings presented here suggest that without subsidisation (e.g., through the PBS), MC poses substantial risks: risk of financial toxicity to patients and their families, and potentially to equitable access to the benefits of RCT participation. Although demonstrated effectiveness is a requirement for pharmaceutical interventions to be listed on the PBS, MC poses a unique scenario where patients are accessing similar interventions covertly through alternative or subaltern therapeutic pathways. There is thus an imperative for commercial entities involved in MC to invest in and support clinical trials to produce high‐quality evidence of efficacy and safety, to ensure quality and to embed equity of future access through registration and subsidisation via the PBS.

The strengths of our study included drawing insights from both those who consented, and those who declined, to participate in an MC trial. An iterative and abductive approach^48^, ^49^, ^50^, ^51^ also foregrounded patients' concerns and critical insights from the study's conceptual framework. The cross‐sectional approach, however, limited data to a single timepoint; the exclusive focus on patients' decision‐making overshadowed carers' perceptions. Future research will further give insights into perceptions and experiences, by purposively sampling patients and carer participants at different trial stages.

## 5 CONCLUSION

The findings and analysis presented here provide novel insights into the perceptions, hopes and concerns of people with advanced cancer regarding the future availability and regulation of MC in Australia. Findings suggest patients are aware and fearful of financial toxicity related to the high cost of accessing MC outside of clinical trials. To improve affordability, equity of access for patients and equity in MC's treatment as a pharmaceutical intervention—interviewees supported making MC a prescription‐only medication subsided by Australia's PBS. Qualitative substudies are valued additions to RCTs—shining light on injustices relevant to RCT recruitment and design, but in this context, policy and practice change may be needed to overcome MC's financial toxicity. Put simply, many interviewees assessed legally available MC to be of little use without ensuring commensurate affordability. Future research could examine the prevalence of concerns in Australia related to MC's financial toxicity and establish the commonality of subaltern or covert use of RC/MC.

## AUTHOR CONTRIBUTIONS

Rebecca E. Olson designed the qualitative substudy, provided oversight on and contributed to data collection and analysis and drafted the final manuscript. Alexandra Smith led the data analysis and was a major contributor in writing the manuscript. Phillip Good contributed to the study design, data analysis and editing of the final version of the manuscript. Morgan Dudley was a major contributor in writing the manuscript. Taylan Gurgenci was a contributor in writing the manuscript and was a major contributor in editing the final version of the manuscript. Janet Hardy contributed to the study design, data analysis and a major contributor in editing the final version of the manuscript. All authors read and approved the final manuscript.

## CONFLICT OF INTEREST

The authors declare no conflict of interest.

## ETHICS STATEMENT

Ethical approval for this study was obtained from the Human Research Ethics Committees at the Mater Hospital (HREC/17/MHS/97) and St Vincent's Hospital (HREC 17/27). All participants provided their written informed consent.

## Data Availability

Data generated and analysed for the current study are available to suitably qualified individuals by request, from the corresponding author, subject to HREC approval.
